# New Medical Journal

**Published:** 1872-02

**Authors:** 


### New Medical Journal.

There comes to us from across the continent an addition to our list of medical exchanges in the form of The Western Lancet, published at San Francisco,

Cal. This journal is published by an association of Physicians who have selected Eustace Trenor, A. M., M. D., and Heman P. Babcock, M. D., as editors. The Journal is divided into six departments, each being under the super_ vision of a separate physician, but under the management of the editor. The number now before ns gives promise of being the first of we hope many very good numbers, and shows the energy and intelligence of our brethren on the Pacifiic coast.

———:o:———

